# Exosomal MicroRNAs in Serum as Potential Biomarkers for Ectopic Pregnancy

**DOI:** 10.1155/2020/3521859

**Published:** 2020-06-11

**Authors:** Jianhua Sun, Gaopi Deng, Xiaofeng Ruan, Si Chen, Huiyan Liao, Xiaorong Liu, Jing Li, Guang Zhao, Jie Gao

**Affiliations:** ^1^The First Clinical Medical College of Guangzhou University of Chinese Medicine, Guangzhou 510000, China; ^2^Lingnan Medical Research Center of Guangzhou University of Chinese Medicine, Guangzhou 510000, China; ^3^Department Obstetrics and Gynecology, First Affiliated Hospital of Guangzhou University of Chinese Medicine, Guangzhou 510000, China; ^4^Tongji Medical College, Huazhong University of Science and Technology, Wuhan 430000, China

## Abstract

**Design:**

From July 2016 to June 2018, 36 women with symptomatic early pregnancy around 4-8 weeks of gestation were recruited into the study. Among them, there were 16 women with viable intrauterine pregnancy (VIP), 9 women with spontaneous abortion (SA), and 11 women of EP. Serum exosomal miRNAs were extracted and measured at the first prenatal visit. Statistical analysis was performed to determine the clinical utility of these biomarkers as single markers and as multimarker panels for EP.

**Results:**

Concentrations of miR-378d in serum exosomes were significantly higher in EP than in VIP and also SA group. As a single marker, miR-378d had the highest specificity of 64% at the sensitivity of 89.1%. Comparatively, both combined panels of hCG, progesterone, miR-100-5p and hCG, progesterone, and miR-215-5P yielded the specificity of 96%. Panels for all markers achieved the highest specificity of 80% at the sensitivity of 91%.

**Conclusions:**

Although further validation in large-scale prospective studies is necessary, our results suggest that serum exosomal miR-378d, miR-100-5p, and miR-215-5P are promising biomarkers for early EP.

## 1. Introduction

Ectopic pregnancy (EP) is defined as a pregnancy that implants outside the uterine cavity as an early complication of pregnancy [1]. 95% of ectopic pregnancies occur in the fallopian tube, where a common occurrence is seen in the ampullary region [2, 3]. EP affects approximately 1% to 2% of all reported pregnant women [4], and despite improvements in detection and treatment [5], it remains the leading cause of pregnancy-related mortality [6, 7]. EPs have been reported to account for as high as 18% among women presenting in the emergency department with first-trimester vaginal bleeding and/or abdominal pain [8]. However, similar symptoms could present in viable intrauterine pregnancies (VIP), as well as spontaneous abortion (SA). Different diagnoses require completely different management. Therefore, accurate and efficient diagnostic methods for early EP are crucial for the appropriate management of symptomatic early pregnancy.

Currently, the diagnosis of EP includes serial transvaginal ultrasonography and serum hCG level measurement [1]. Progesterone alone is not a biomarker for ectopic pregnancy [9]. EP can be diagnosed definitely by ultrasonography when a gestational sac with a yolk sac or/and embryo in the adnexa is detected [10, 11]. However, some of the ectopic pregnancies are just too early and too small to be visualized in the initial ultrasound examination [12]. A single hCG concentration assay is not efficient to identify the location or viability of a gestation. Serial hCG level measurements are often used to distinguish between normal and abnormal pregnancies in the clinic [13–15]. Patients may be at risk of tubal rupture before the next hCG measurement and ultrasound scan. Taken together, using ultrasonography, hCG and progesterone are limited and insufficient to identify the location of the pregnancy at an early stage. Therefore, it is critical to develop additional noninvasive markers for the diagnosis of EP in the early pregnancy stage.

In this research, we only assay exosomes in relation to pregnancy. Exosomes are small membrane vesicles (30-100 nm in size) [16] of endocytic origin that contain diverse biomolecules, such as RNAs (including microRNAs), specific sets of lipids, and proteins. Their cargo can reflect the physiological state of the original cells and also regulate the recipient cells. Hence, exosomes might be used as biomarkers for early diagnosis of various human diseases [17]. MicroRNAs (miRNAs), small noncoding RNAs, suppress target function via sequence-specific interactions with the mRNA 3′-untranslated region (3′-UTR) [18] which is highly stable [19]. Exosomes are abundant in miRNAs available for potential biomarker profiling, being protected by RNases as compared to intracellular miRNAs or miRNAs in cell-free blood [20]. In the process of physiological normal early pregnancy which is 6 weeks, exosomes of trophoblast origin can be detected from the week of gestation in the maternal circulation. The number of the vesicles increases until it changes to normal level in 48 hours after delivery [21]. The level of placenta-specific exosomes and their content and information are likely to affect placental health. Placental dysfunction leads to the increasing shedding of trophoblast-derived extracellular vesicles into the maternal circulation and releases of toxic material subsequently including antiangiogenic factors, proinflammatory mediators, and microRNAs [22]. Studies on exosomal biomarkers have been carried out in many diseases such as cancer, exosomes are also gaining interest in pregnancy-associated complications such as hypertension and preeclampsia [21–23]. Nevertheless, exosomes have not been investigated for prediction of early pregnancy outcomes.

In this study, we aim to identify exosomal miRNAs and their changes in serum levels in EP and to explore their predictive value for the identification of EP.

## 2. Materials and Methods

### 2.1. Study Design and Sample Collection

A total of 36 women who presented to Guangzhou University of Chinese Medicine with symptomatic early pregnancy were included in this study. Data were collected using a convenient follow-up system supported by Empower Electronic Data Capture (EDC) system (Solutions, Shanghai, China). All samples were collected after the informed consent was signed. The study was approved by the Institutional Review Board (No. [2014] 050) and has been registered at the Chinese Clinical Trial Registry, http://www.chictr.org.cn (ChiCTR1900021892).

Remnant serum specimens, which were sent to the laboratory from the emergency department for physician-ordered hCG testing between July 2016 and June 2018, were obtained. Women who were included in this study need to meet the following criteria: (1) serum hCG > 5 IU/L, (2) complaint of vaginal bleeding and abdominal pain, (3) sufficient serum available for progesterone and exosomal miRNA testing, (4) aged ≥ 18 years, (5) gestational age by the last menstrual period (GA) ≤ 8 weeks, (6) natural pregnancy, and (7) singleton gestation. Women were excluded if they had (1) any management before enrollment during this pregnancy, (2) nontubal ectopic pregnancy, (3) any evidence of gestational trophoblastic disease, or (4) multiple pregnancies.

The patients were followed up prospectively until pregnancy outcomes were determined; this was based on an internationally recognized nomenclature [11]. Viable intrauterine pregnancy (VIP) was defined as the detection of a gestational sac with a yolk sac or embryo in the uterine cavity by ultrasound. Spontaneous abortion (SA) was defined as conception if serum hCG declined spontaneously at <5 mIU/mL or if the woman had an empty sac, embryonic demise, or retained trophoblast tissue. Ectopic pregnancy (EP) was defined as ultrasound or laparoscopic findings of an adnexal mass with evidence of elevated serum hCG levels after uterine evacuation. 16 women with VIP, 11 women with EP, and 9 women with SA were included if they met all of the above inclusive criteria.

We identified miRNAs (miR-100-5p, miR-122-5p, miR-146a-5p, miR-184, miR-215-5p, and miR-378d) differentially and expressed the serum exosomes in pregnant women with EP, SA, and VIP using miRNA sequencing.

Maternal age, parity, gestational age, and initial concentration of hCG were extracted and validated by Excel on presentation. The pregnancy outcome of patients was validated by using chart abstraction when necessary. Blood specimens were collected from patients' initial presentation using tubes not containing ethylenediaminetetraacetic acid (EDTA), centrifuged at 4000g for 10 minutes, split into 0.5 mL aliquots, and frozen in -80°C for less than 2 years before exosomal miRNAs and progesterone testing was performed.

### 2.2. Exosome Isolation and Identification, Exosomal RNA Extraction, Preparation of Sequencing Library RT-qPCR, and hCG and Progesterone Testing

#### 2.2.1. Exosome Isolation and Identification

Exosomes were isolated from 800 *μ*L serum using the Ribo exosome isolation reagent (for plasma or serum) as instructed by the manufacturer. Briefly, serum samples were centrifuged at 2000 × g for 20 minutes to remove residual cells and debris. Next, 1/3 volumes of exosome isolation reagent were added to the supernatant and the specimens were incubated at 4°C for 30 min before precipitation by centrifugation at 15000 × g for 2 minutes. Then, the exosome precipitate was obtained and used immediately. We used a transmission electron microscope (FEI TECNAI G2; FEI Ltd., Hillsboro, America) and NanoSight (ZETASIZER Nano series-Nano-ZS, Malvern) to detect the ultrastructure and particle size distribution of the exosomes, respectively. The protein markers CD63 (BD) and CD81 (CD81-Antibody-FITC, BD #551108, 100 tests) were stained and confirmed by using flow cytometry (Accuri C6 flow cytometer) (see Supplementary Figure S1).

#### 2.2.2. Exosomal RNA Extraction

Exosomal total RNAs containing miRNAs were extracted from 800 *μ*L serum using the Magzol™ Reagent and HiPure Serum/Plasma miRNA Kit (Magen™) according to the manufacturer's protocol. Total RNA was eluted with 10 *μ*L of RNase-free water. The quality and quantity of RNA were determined by K5500 microspectrophotometer UV (K.O.) with the manufacturer's instructions.

#### 2.2.3. Real-Time Quantitative Reverse Transcriptase Polymerase Chain Reaction Analysis of miRNAs

Significant differential expressions for miRNAs (hsa-miR-100-5p, hsa-miR-122-5p, hsa-miR-146a-5p, hsa-miR-184, hsa-miR-215-5p, and hsa-miR-378d) of the three groups were measured by using qRT-PCR. qRT-qPCR was performed on miDETECT A Track™ miRNA qRT-PCR Starter Kit (RiboBio) in 96-well plates using Primer (Applied Biosystems, Foster City, CA, USA). To compare the concentrations of exosomal miRNA in the serum, we normalized the data from *Caenorhabditis elegans* miR-39 (cel-miR-39; RiboBio), which was added to the specimens to normalize the serum volume at the beginning of RNA extraction, as described previously [24]. The bulge-loop qRT-PCR primers and cel-miR-39 were purchased from RiboBio. The PCR amplification was performed at 95°C for 20 s, followed by 40 cycles at 95°C for 10 s, 60°C for 30 s, and 70°C for 10 s. A dA dissociation curve was analyzed from 70°C to 95°C. The relative expression levels of exosomal miRNAs were calculated using the comparative 2^-*ΔΔ*Ct^ method. All reactions were run in triplicate.

#### 2.2.4. hCG and Progesterone Testing

Serum levels of hCG and progesterone were measured at the First Affiliated Hospital of Guangzhou University of Chinese Medicine using a commercially available assessment from licensed medical technicians blinded to miRNA results and pregnancy outcomes. Serum hCG was tested using the hCG+*β* kit (Roche) and serum progesterone from the progesterone III kit (Roche) by fully automated electrochemiluminescence immunoassay system (Roche Cobas e 601) in accordance with the manufacturer's instructions. The hCG measurement has a dynamic range of 0.1-10000 IU/L and a repeatability of CV of 4.4%, 1.3%, and 1.7% for quality control samples with means of 4.81, 880, and 7949 IU/L, respectively. The progesterone measurement has a dynamic range of 0.05-60 ng/mL (0.159-191 nmol/L) and an interassay CV of 5.3%, 3.7%, and 2.9% for quality control samples with means of 2.35, 4.68, and 28.5 nmol/L (0.739, 1.47, and 8.96 ng/mL), correspondingly.

### 2.3. Statistical Analysis

Baseline characteristics were compared between EP and non-EP (SA/VIP) groups. Continuous variables were assessed using Mann-Whitney *U* test. Fisher's exact test was applied for comparison of sensitivity, and categorical variables were used where appropriate. Receiver operating characteristic (ROC) curves for both a single biomarker and multiple biomarkers discriminating pregnancy outcomes were plotted, and areas under the curves (AUC) were calculated. Since we established the diagnostic model in this study focusing on screening patients for ectopic pregnancy, the corresponding specificity was analyzed. Due to the small sample size, we used TYPE 1B: development and validation using resampling (2000 times, bootstrap) in compliance with the TRIPOD guidelines. All of the statistical analyses were performed with the R statistical software (https://www.r-project.org) and EmpowerStats (https://www.r-project.org, X&Y Solutions, Inc., Boston, MA). *P* < 0.05 was considered statistically significant.

## 3. Results

### 3.1. Baseline Characteristics

Thirty-six patients were included in the analysis. There were 11 EPs, 9 SAs, and 16 VIPs after outcome verification. Baseline characteristics are shown in Table 1. There was no significant difference in maternal age and gestational age among these three groups. There was also no statistically significant difference in serum hCG, circulating exosomal miR-122-5P, and miR-378d among the three groups. The levels of serum exosomal miR-215-5P and serum progesterone in the EP group were statistically the lowest in these three groups, while the levels of serum exosomal miR-100-5P, miR-146a-5P, and miR-184 in the EP group were statistically lower compared with the VIP group, but were higher compared with the SA group (see Table 1).

### 3.2. Diagnostic Value of Exosomal MicroRNAs in Serum

Table 2 shows the area under the curve (AUC) for all individual markers and different multimarker combinations. The ROC curves were constructed for differentiating EP and SA and VIP based on serum concentrations of exosomal miRNAs, hCG, and progesterone using a single marker and combination in test.

**Figure 1:**
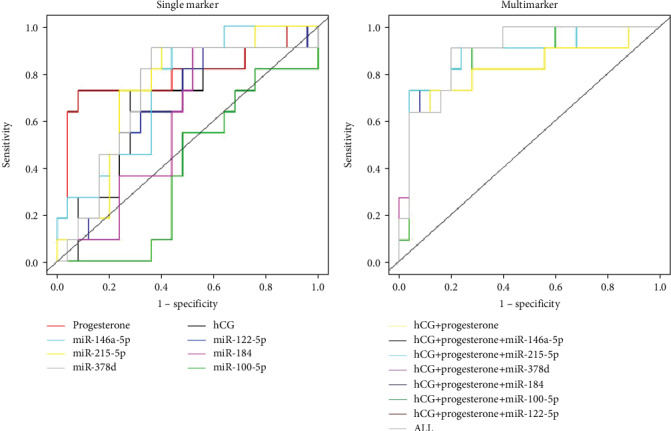
ROC analysis of single markers and multimarkers for discriminating EP from SA/VIP.

As single marker, progesterone had the highest AUC of 0.79 (95% confidence interval (CI), 0.599-0.99). Some of the miRNAs in serum exosomes (Table 2), miR-378d, in the EP group yielded relatively high AUC of 0.72 (95% confidence interval (CI), 0.52-0.91) compared with those in the other groups. The AUCs of miR-215-5p and miR-146a-5p in the EP group were higher than those in the SA group, while lower than those in the VIP group (0.73, 95% confidence interval (CI), 0.56-0.91; 0.73, 95% confidence interval (CI), 0.57-0.91), whereas miR-378d had the highest specificity of 64% over than all other miRNAs. miR-215-5p and miR-146a-5p showed a specificity of 60% and 56%, respectively (see Table 2 and Figure 1).

To evaluate the multimarker diagnostic performance added by the inclusion of miRNAs, we conducted a multivariate logistic regression to detect the value of different combinations of biomarkers. The AUCs of different multimarker panels ranged from 0.81 to 0.89 for detecting EP (Table 2). Both the combination panel of hCG+progesterone+miR-100-5p and hCG+progesterone+miR-215-5P achieved the specificity of 96%, which is 8% higher than that of combination panel of hCG+progesterone. The combination of other miRNAs from hCG+progesterone panel did not add the specificity. Combined hCG, progesterone, and all six miRNAs had the highest specificities of 80% at the sensitivity of 91% (see Table 2 and Supplementary Table S1).

The diagnostic value of single marker and multimarker to detect EP from SA and VIP was performed as well. For single markers, hCG and progesterone yielded AUCs of 0.67 and 0.79. With AUCs of 0.73 and 0.72, miR-378d and miR-215-5P had the highest specificity of 60% and 64%. The AUCs of different multimarker panels ranged from 0.81 to 0.89 for discriminating EP from SA and VIP.

## 4. Discussion

Knowledge of pregnancy outcomes for individual patient in an early stage has played an important role because different pregnancy outcomes result in different therapeutic choices in first-trimester symptomatic pregnancies. In this study, we used RNA sequencing to detect novel exosomal miRNAs, which might be useful for predicting EP. We selected 6 miRNAs differently and expressed in the EP, VIP, and SA groups; we tested the concentrations of circulating exosomal miRNAs using RT-qPCR and observed a significant relation of exosomal miRNA concentrations with early pregnancy outcomes through the follow-up. The serum concentrations of exosomal miR-146a-5p, miR-215-5p, and miR-378d were statistically different between women with EP and women with VIP/SA. Our results showed decreased levels of miR-146a-5p, miR-215-5p, and miR-378d in serum exosome samples from women with EP compared with serum exosome samples from women with SA/VIP. Our data suggested that circulating exosomal miRNAs may serve as a sensitive biomarker for the predicting EP in early pregnancy with vaginal bleeding or abdominal pain.

As a single marker for detecting EP, both hCG and progesterone in our study achieved similar result of AUCs to the study conducted by Ye et al., with even lower specificity at 90% sensitivity [25], which indicated that hCG or progesterone alone was not sufficient to diagnose EP. miR-146a-5p achieved an AUC of 0.73, which was equal to that of circulating miR-323-3p, while the specificity was 12.4% greater than miR-323-3p [25]. Multimarker panels for hCG+progesterone+miR-146a-5p had a high AUC of 0.88 with 76% specificity and 91% specificity, respectively. While the combination of all markers yields the highest AUC of 0.89 with the highest specificity at high sensitivity, it is an expensive and not an ideal biomarker panel for clinical use [25].

An ideal marker for the diagnosis of EP would be the one that can distinguish EP from SA. One previous report about circulating concentrations of miRNAs as biomarkers of EP showed that miR-323-3p was highly expressed in women with EP compared to women with VIP/SA [25]. Another study about plasma miRNAs for detecting EP, including 18 EPs, 12 SAs, and 26 VIPs, demonstrated that miR-323-3p yielded an AUC of 0.7454 for discriminating EP from SA [26]. In our study, however, multiple panels of hCG+progesterone+miR-146a-5p performed better than a single marker in distinguishing EP from SA with an AUC of 0.88.

Diagnosis of EP at an early stage is crucial because several optimal treatments can be used to control EP progression. Over 20 markers for detecting EP have been studied, but the clinical application of these markers was still limited. Thus, it is critical to investigate new biomarkers to predict EP to help make an appropriate clinical decision. To the best of our best knowledge, this is the first time to prove the potential clinical significance of serum exosomal miRNAs for the detection of EP in an early vaginal bleeding/abdominal pain cohort. Exosomes, containing proteins, nucleic acids, and lipids, play a critical role in intercellular communication and are promising diagnostic or prognostic biomarkers for clinical application [26]. Circulating exosomal miRNAs as promising diagnostic, therapeutic, and prognostic biomarkers have recently emerged for many malignancies, and exosomes in pregnancy-related complications have been studied as well [27, 28]. In this study, we detected novel promising predicting biomarkers from patients' serum specimens to improve the prediction of early EP, and we found that exosomal miR-146a-5p is a potential promising marker.

Investigations of these differentially expressed miRNAs in pregnancy-associated disease, however, are still limited. A previous study showed circulating extracellular miR-215-5p in women of the second trimester was determined in association with birth weight-at-gestational age [29]. Another study showed that miR-146a-5p was significantly overexpressed in trophoblast cellular and exosomes of pregnancy with antiphospholipid antibody (aPL), an increased risk of pregnancy complications. It indicated that miR-146a-5p may play a role in aPL-induced trophoblast inflammation [30]. But in intrauterine growth restriction pregnancies, miR-146a-5p was downregulated in the maternal circulation [31]. miR-100-5p in maternal whole peripheral blood was found downregulated in intrauterine growth restriction, gestational hypertension, and preeclampsia [29]. To our knowledge, there has not been a reference for miRNA-378d in pregnancy-associated disease and miR-184 was found overexpressed in both villi and decidua tissues of recurrent spontaneous abortion patients [32]. However, there is not enough work on the relationship of these miRNAs with early pregnancy outcomes. These confirmed that differentially expressed exosomal miRNAs may provide new insights into the physiology and pathology in early pregnancy.

In this study, we used cel-miR-39 (an exogenous reference gene) for normalization instead of RNU6B. Dong et al. demonstrated that for the quantification of circulating microRNAs, U6 was not suitable as an endogenous control, particularly in frozen-thawed samples [33]. Synthetic miRNAs added at the point of denaturation of plasma or serum helped to avoid different efficiencies of RNA extraction due to various content of protein and lipid in samples [33]. Many studies have used cel-miR-39 to normalize exosomal miRNA expression [34, 35]. Our study shows high concordance for replicates and across plates of each specimen, and the data variability has been corrected by normalization based on cel-miR-39.

Indeed, extracellular miRNAs have emerged as attractive biomarkers because they are noninvasive and have been examined as predictive tools in many pregnancy-associated complications [36, 37], including EP [25, 38]. Their reproducible concentrations in circulation across many individuals and intrinsic stability make miRNAs attractive biomarkers [33]. Exosomes can protect their cargos (including miRNAs) from damage or clearance because of their bilayer membrane and nano size, extending their circulation half-life and improving their bioactivity [26]. Moreover, many peripheral blood miRNAs are released from necrotic and apoptotic cells passively and may not truly illustrate the biological state of the original cells [19, 38]. In contrast, exosomal miRNAs are secreted in the peripheral circulation by various cell types actively and consequently represent a more authentic physiological status of related tissues through miRNA transfer [26]. Hereafter, exosomal miRNAs may be truly representative of specific molecular biomarkers, compared with cell-free miRNAs. On the other hand, although the pathophysiology of EP is still unknown, it is generally believed that EP is a consequence of altered trophoblast invasion. This phenomenon is attributed to injured trophoblast invasion and subsequent release of possibly harmful materials including extracellular vesicles. More than one thousand miRNAs are expressed by different layers of the human placenta. The function of placentally expressed miRNAs is not fully elucidated but is clear that they take part in the regulation of placental development and are essential for normal physiology. All of the abovementioned mechanisms are associated with the characteristics of PE, such as reduced trophoblast invasion, angiogenesis in vitro. Unfortunately, no research had been conducted on the accurate mechanism between EP and miRNAs, which needs further research.

The imprecision we detected in the prediction of early pregnancy outcomes with exosomal miRNAs may be caused by some limitations of this study. One limitation is the small sample size, so larger research is needed to determine whether miR-146a-5p in serum exosomes is a universal biomarker for the diagnosis of EP. Another limitation is that the functional significance of these exosomal miRNAs in pregnancy has not been illustrated, but it provides ideas about the role of circulating exosomal miRNAs in early pregnancy for future research.

Despite these limitations, our study has advantages such as the use of second-generation sequencing to select novel biomarkers in circulating exosomes. Additionally, blind methods were adopted. Workers collecting samples and technicians measuring hCG, progesterone, and miRNAs were blinded to pregnancy outcomes. Technicians for miRNA assay and technicians for hCG and progesterone assay are also blinded to each other in terms of respective results. Moreover, it is noteworthy that this study was performed on women with many earlier symptomatic pregnancies and it would be helpful for clinicians' decision on the timely intervention of symptomatic pregnancies.

In conclusion, we verified the clinical significance of pregnancy-associated miRNAs (miR-378d, miR-100-5p, and miR-215-5P) in serum exosomes as potential biomarkers for the prediction of ectopic pregnancy. Circulating levels of exosomal pregnancy-associated miRNAs in serum can distinguish EP/SA from VIP with high specificity. Compared with the combination of hCG and progesterone, the addition of serum exosomal miR-100-5p or miR-215-5P can discriminate EP from SA with higher specificity. Although our study is still preliminary due to the small sample size, the assay of exosomal miRNAs in serum has potential as a predicting test for EP. Future large-scale studies of exosomal miRNAs in the serum of early pregnancy singly or in combination with other biomarkers may contribute to developing an accurate test for the diagnosis of early ectopic pregnancy.

## 5. Conclusions

Although further validation in large-scale prospective studies is necessary, our results suggest that serum exosomal miR-378d, miR-100-5p, and miR-215-5P are promising biomarkers for early EP.

## Figures and Tables

**Table 1:** General characteristics of women in EP vs. SA vs. VIP.

Characteristic	EP	SA	VIP	*P* value∗
*N*	11	9	16	
Age	33.00 (31.00-36.00)	30.00 (27.00-38.00)	30.50 (27.00-32.00)	0.250
GA (wk)	6.14 (5.64-6.57)	6.57 (6.29-6.86)	5.29 (4.82-6.71)	0.131
Progesterone	34.52 (16.41-62.62)	66.30 (60.34-106.10)	75.29 (64.49-85.19)	0.023
hCG	6551 (1546-12780)	7828 (7213-36696)	6872 (1885-56301)	0.141
miR-100-5P	0.69 (0.31-1.16)	0.25 (0.07-0.51)	2.00 (0.57-5.97)	0.048
miR-122-5P	0.48 (0.32-1.51)	0.50 (0.28-3.59)	4.02 (0.87-83.26)	0.052
miR-146a-5P	1.54 (0.71-1.85)	1.39 (0.86-4.80)	9.21 (1.82-29.42)	0.025
miR-184	1.38 (0.54-2.25)	0.45 (0.42-0.99)	4.32 (1.57-31.99)	0.031
miR-215-5P	2.74 (1.85-4.38)	5.77 (1.57-8.52)	17.52 (5.62-186.69)	0.027
miR-378d	2.24 (1.35-5.98)	6.62 (2.88-11.16)	36.14 (8.84-114.33)	0.059

EP: ectopic pregnancy; SA: spontaneous abortion; VIP: viable intrauterine pregnancy; GA: gestational age. Nonnormal distribution of data was presented as median (interquartile range). *P* < 0.05 was considered statistically significant. ^∗^Mann-Whitney *U* test.

**Table 2:** Diagnostic value of the single marker and multimarker panels to predict EP or SA or VIP in early symptomatic pregnancies.

Characteristic	EP			SA			VIP		
	AUC (95% CI)	Sen.	Spe.	AUC (95% CI)	Sen.	Spe.	AUC (95% CI)	Sen.	Spe.
Progesterone	0.79 (0.59-0.99)	0.73	0.92	0.41 (0.18-0.64)	0.56	0.52	0.68 (0.50-0.86)	0.99	0.40
hCG	0.67 (0.48-0.86)	0.73	0.72	0.69 (0.50-0.88)	0.89	0.63	0.49 (0.29-0.70)	0.13	0.99
miR-100-5P	0.39 (0.20-0.58)	0.55	0.52	0.69 (0.45-0.93)	0.78	0.70	0.74 (0.57-0.90)	0.81	0.60
miR-122-5P	0.67 (0.47-0.87)	0.91	0.44	0.62 (0.40-0.85)	0.67	0.63	0.74 (0.57-0.91)	0.81	0.65
miR-146a-5P	0.73 (0.57-0.90)	0.91	0.56	0.57 (0.35-0.79)	0.56	0.70	0.75 (0.58-0.93)	0.75	0.75
miR-184	0.58 (0.38-0.78)	0.91	0.48	0.73 (0.51-0.95)	0.78	0.74	0.74 (0.57-0.92)	0.63	0.85
miR-215-5P	0.73 (0.56-0.91)	0.91	0.60	0.44 (0.21-0.66)	0.67	0.41	0.75 (0.58-0.92)	0.69	0.85
miR-378d	0.72 (0.52-0.91)	0.91	0.64	0.54 (0.34-0.74)	0.89	0.44	0.72 (0.52-0.91)	0.75	0.85
hCG+P	0.81 (0.63-0.99)	0.73	0.88	0.63 (0.39-0.86)	0.56	0.78	0.67 (0.48-0.85)	0.88	0.55
hCG+P+miR-100-5P	0.87 (0.74-0.99)	0.73	0.96	0.69 (0.50-0.88)	0.89	0.52	0.79 (0.64-0.94)	0.88	0.65
hCG+P+miR-122-5P	0.82 (0.64-0.99)	0.73	0.92	0.68 (0.48-0.88)	0.89	0.48	0.70 (0.52-0.88)	0.94	0.45
hCG+P+miR-146a-5P	0.88 (0.75-0.99)	0.91	0.76	0.64 (0.41-0.87)	0.75	0.56	0.78 (0.62-0.94)	0.88	0.70
hCG+P+miR-184	0.82 (0.64-0.99)	0.73	0.92	0.67 (0.47-0.88)	0.89	0.52	0.68 (0.50-0.86)	0.81	0.65
hCG+P+miR-215-5P	0.88 (0.74-0.99)	0.73	0.96	0.68 (0.48-0.88)	0.89	0.52	0.78 (0.62-0.93)	0.94	0.55
hCG+P+miR-378d	0.82 (0.64-0.99)	0.73	0.88	0.64 (0.42-0.86)	0.56	0.81	0.69 (0.51-0.87)	0.81	0.65
ALL∗	0.89 (0.79-0.99)	0.91	0.80	0.69 (0.5-0.88)	0.78	0.59	0.82 (0.69-0.96)	0.99	0.60

Sen: sensitivity; Spe: specificity; P: progesterone; ∗ALL = hCG+progesterone+miR-100-5P+miR-122-5P+miR-146a-5P+miR-184+miR-215-5P+miR-378d.

## Data Availability

The data used to support the findings of this study are available from the corresponding author upon request.

## Supplementary Materials

Supplementary 1 The details of exosome isolation and identification.

Supplementary 2 Positive predictive value and negative predictive value of multimarker panels to predict EP or SA or VIP in early symptomatic pregnancies.

## References

[1] Committee on Practice Bulletins—Gynecology (2018). ACOG Practice Bulletin No. 191: tubal ectopic pregnancy. *Obstetrics & Gynecology*.

[2] Bouyer J., Coste J., Fernandez H., Pouly J. L., Job-Spira N. (2002). Sites of ectopic pregnancy: a 10 year population-based study of 1800 cases. *Human Reproduction*.

[3] Shaw J. L. V., Dey S. K., Critchley H. O. D., Horne A. W. (2010). Current knowledge of the aetiology of human tubal ectopic pregnancy. *Human Reproduction Update*.

[4] Centers for Disease Control and Prevention (CDC) (1995). Ectopic pregnancy--United States, 1990-1992. *Morbidity and Mortality Weekly Report*.

[5] Hoover K. W., Tao G., Kent C. K. (2010). Trends in the diagnosis and treatment of ectopic pregnancy in the United States. *Obstetrics and Gynecology*.

[6] Creanga A. A., Syverson C., Seed K., Callaghan W. M. (2017). Pregnancy-related mortality in the United States, 2011-2013. *Obstetrics and Gynecology*.

[7] Creanga A. A., Shapiro-Mendoza C. K., Bish C. L., Zane S., Berg C. J., Callaghan W. M. (2011). Trends in ectopic pregnancy mortality in the United States. *Obstetrics and Gynecology*.

[8] Murray H., Baakdah H., Bardell T., Tulandi T. (2005). Diagnosis and treatment of ectopic pregnancy. *Canadian Medical Association Journal*.

[9] Davies M. (2013). Cassandra's prophecy: response on behalf of the Royal College of Obstetricians and Gynaecologists. *Reproductive BioMedicine Online*.

[10] Barnhart K. T., Fay C. A., Suescum M. (2011). Clinical factors affecting the accuracy of ultrasonography in symptomatic first-trimester pregnancy. *Obstetrics and Gynecology*.

[11] Barnhart K., van Mello N. M., Bourne T. (2011). Pregnancy of unknown location: a consensus statement of nomenclature, definitions, and outcome. *Fertility and Sterility*.

[12] Kirk E., Daemen A., Papageorghiou A. T. (2008). Why are some ectopic pregnancies characterized as pregnancies of unknown location at the initial transvaginal ultrasound examination?. *Acta Obstetricia et Gynecologica Scandinavica*.

[13] Seeber B. E., Sammel M. D., Guo W., Zhou L., Hummel A., Barnhart K. T. (2006). Application of redefined human chorionic gonadotropin curves for the diagnosis of women at risk for ectopic pregnancy. *Fertility and Sterility*.

[14] Morse C. B., Sammel M. D., Shaunik A. (2012). Performance of human chorionic gonadotropin curves in women at risk for ectopic pregnancy: exceptions to the rules. *Fertility and Sterility*.

[15] Barnhart K. T., Sammel M. D., Rinaudo P. F., Zhou L., Hummel A. C., Guo W. (2004). Symptomatic patients with an early viable intrauterine pregnancy. *Obstetrics and Gynecology*.

[16] Mathivanan S., Ji H., Simpson R. J. (2010). Exosomes: extracellular organelles important in intercellular communication. *Journal of Proteomics*.

[17] Pant S., Hilton H., Burczynski M. E. (2012). The multifaceted exosome: biogenesis, role in normal and aberrant cellular function, and frontiers for pharmacological and biomarker opportunities. *Biochemical Pharmacology*.

[18] Bartel D. P. (2009). MicroRNAs: Target Recognition and Regulatory Functions. *Cell*.

[19] Cortez M. A., Bueso-Ramos C., Ferdin J., Lopez-Berestein G., Sood A. K., Calin G. A. (2011). MicroRNAs in body fluids--the mix of hormones and biomarkers. *Nature Reviews Clinical Oncology*.

[20] Cheng L., Sharples R. A., Scicluna B. J., Hill A. F. (2014). Exosomes provide a protective and enriched source of miRNA for biomarker profiling compared to intracellular and cell-free blood. *Journal of Extracellular Vesicles*.

[21] Salomon C., Torres M. J., Kobayashi M. (2014). A gestational profile of placental exosomes in maternal plasma and their effects on endothelial cell migration. *PLoS One*.

[22] Yáñez-Mó M., Siljander P. R. M., Andreu Z. (2015). Biological properties of extracellular vesicles and their physiological functions. *Journal of Extracellular Vesicles*.

[23] Bhagirath D., Yang T. L., Bucay N. (2018). microRNA-1246 is an exosomal biomarker for aggressive prostate cancer. *Cancer Research*.

[24] Salomon C., Guanzon D., Scholz-Romero K. (2017). Placental exosomes as early biomarker of preeclampsia: potential role of exosomal microRNAs across gestation. *The Journal of Clinical Endocrinology and Metabolism*.

[25] Ye S. B., Zhang H., Cai T. T. (2016). Exosomal miR-24-3p impedes T-cell function by targetingFGF11and serves as a potential prognostic biomarker for nasopharyngeal carcinoma. *The Journal of Pathology*.

[26] Senapati S., Barnhart K. T. (2013). Biomarkers for ectopic pregnancy and pregnancy of unknown location. *Fertility and Sterility*.

[27] Miura K., Higashijima A., Mishima H. (2015). Pregnancy-associated microRNAs in plasma as potential molecular markers of ectopic pregnancy. *Fertility and Sterility*.

[28] He C., Zheng S., Luo Y., Wang B. (2018). Exosome theranostics: biology and translational medicine. *Theranostics*.

[29] Huang X., Liang M., Dittmar R., Wang L. (2013). Extracellular microRNAs in urologic malignancies: chances and challenges. *International Journal of Molecular Sciences*.

[30] Mitchell M. D., Peiris H. N., Kobayashi M. (2015). Placental exosomes in normal and complicated pregnancy. *American Journal of Obstetrics and Gynecology*.

[31] Rodosthenous R. S., Burris H. H., Sanders A. P. (2017). Second trimester extracellular microRNAs in maternal blood and fetal growth: an exploratory study. *Epigenetics*.

[32] Hromadnikova I., Kotlabova K., Hympanova L., Krofta L. (2016). Gestational hypertension, preeclampsia and intrauterine growth restriction induce dysregulation of cardiovascular and cerebrovascular disease associated microRNAs in maternal whole peripheral blood. *Thrombosis Research*.

[33] Dong F., Zhang Y., Xia F. (2014). Genome-wide miRNA profiling of villus and decidua of recurrent spontaneous abortion patients. *Reproduction*.

[34] Xiang M., Zeng Y., Yang R. (2014). U6 is not a suitable endogenous control for the quantification of circulating microRNAs. *Biochemical and Biophysical Research Communications*.

[35] Kroh E. M., Parkin R. K., Mitchell P. S., Tewari M. (2010). Analysis of circulating microRNA biomarkers in plasma and serum using quantitative reverse transcription-PCR (qRT-PCR). *Methods*.

[36] Qin X., Yu S., Zhou L. (2017). Cisplatin-resistant lung cancer cell–derived exosomes increase cisplatin resistance of recipient cells in exosomal miR-100–5p-dependent manner. *International Journal of Nanomedicine*.

[37] Li J., Tan M., Xiang Q., Zhou Z., Yan H. (2017). Thrombin-activated platelet-derived exosomes regulate endothelial cell expression of ICAM-1 via microRNA-223 during the thrombosis-inflammation response. *Thrombosis Research*.

[38] Anton L., Olarerin-George A. O., Schwartz N. (2013). miR-210 inhibits trophoblast invasion and is a serum biomarker for preeclampsia. *The American Journal of Pathology*.

