# Supplementary figures and images for: Exosomal MicroRNAs in Serum as Potential Biomarkers for Ectopic Pregnancy
Source: Biomed Res Int. 2020 Jun 11;2020:3521859. doi: 10.1155/2020/3521859 (PMC7305552; doi:10.1155/2020/3521859)

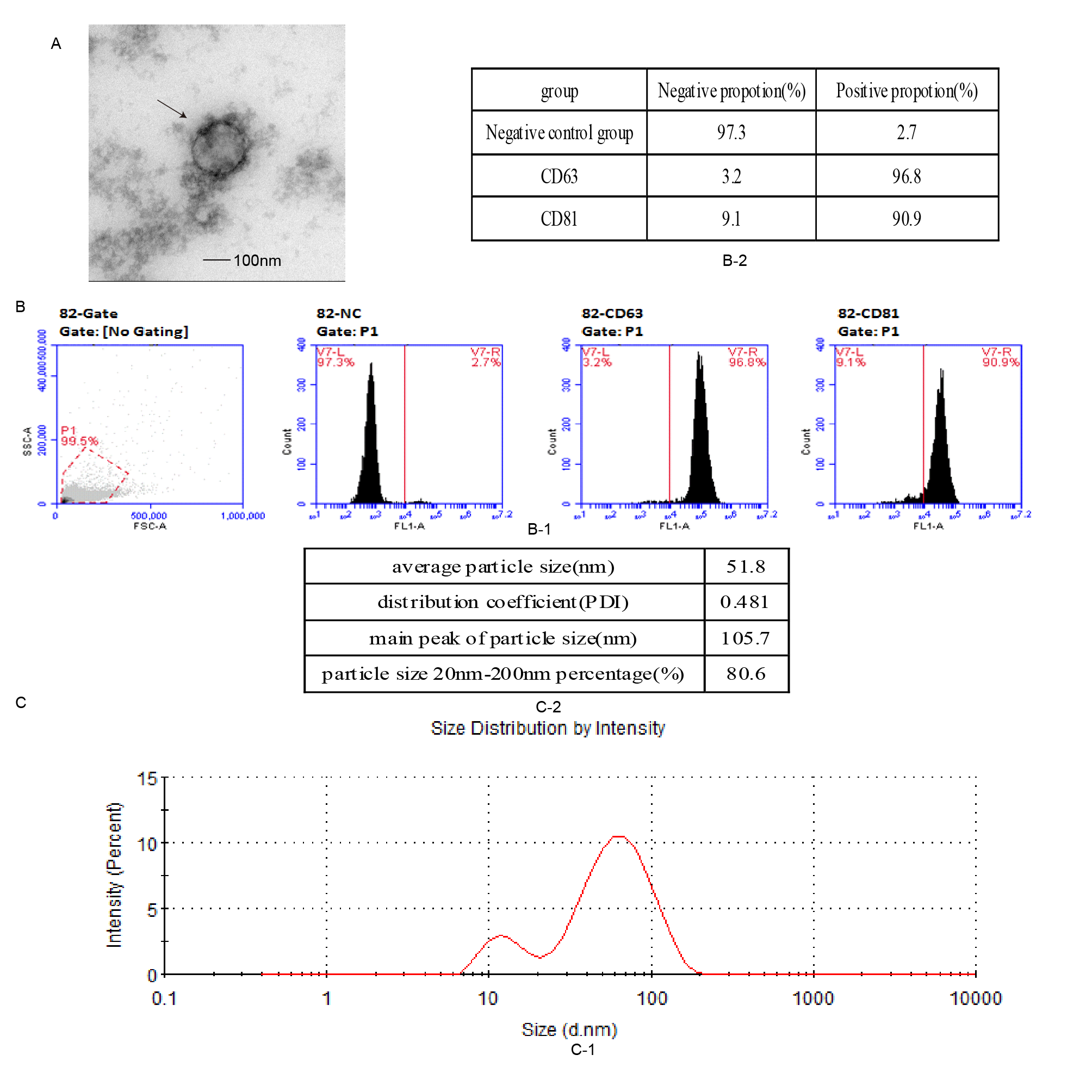

Supplement: Supplementary 1 — The details of exosome isolation and identification. [file 3521859.f1.tif]
