# Supplementary material for: Exosomal MicroRNAs in Serum as Potential Biomarkers for Ectopic Pregnancy
Source: Biomed Res Int. 2020 Jun 11;2020:3521859. doi: 10.1155/2020/3521859 (PMC7305552; doi:10.1155/2020/3521859)
Supplement: Supplementary 2 — Positive predictive value and negative predictive value of multimarker panels to predict EP or SA or VIP in early symptomatic pregnancies. [file 3521859.f2.pdf]

Table S1: Positive Predictive Value and Negative Predictive Value of multimarker panels to predict EP or SA or VIP in early symptomatic pregnancies.

Characteristic	EP			SA			VIP		
	AUC（95%CI）	PPV	NPV	AUC（95%CI）	PPV	NPV	AUC（95%CI）	PPV	NPV
HCG+P	0.81（0.63-0.99）	0.73	0.88	0.63（0.39-0.86）	0.45	0.84	0.67（0.48-0.85）	0.61	0.85
HCG+P+ miR-100-5P	0.87（0.74-0.99）	0.89	0.89	0.69（0.50-0.88）	0.38	0.93	0.79（0.64-0.94）	0.67	0.87
HCG+P+ miR-122-5P	0.82（0.64-0.99）	0.80	0.88	0.68（0.48-0.88）	0.36	0.93	0.70（0.52-0.88）	0.58	0.90
HCG+P+ miR-146a-5P	0.88（0.75-0.99）	0.63	0.95	0.64（0.41-0.87）	0.50	0.85	0.78（0.62-0.94）	0.70	0.88
HCG+P+ miR-184	0.82（0.64-0.99）	0.80	0.88	0.67（0.47-0.88）	0.38	0.93	0.68（0.50-0.86）	0.65	0.81
HCG+P+ miR-215-5P	0.88（0.74-0.99）	0.89	0.89	0.68（0.48-0.88）	0.38	0.93	0.78（0.62-0.93）	0.63	0.92
HCG+P+ miR-378d	0.82（0.64-0.99）	0.73	0.88	0.64（0.42-0.86）	0.50	0.85	0.69（0.51-0.87）	0.65	0.81
ALL*	0.89（0.79-0.99）	0.67	0.95	0.69（0.5-0.88）	0.39	0.89	0.82（0.69-0.96）	0.67	0.99

PPV: Positive Predictive Value; NPV: Negative Predictive Value; P: progesterone; *ALL= HCG+ progesterone + miR-100-5P+ miR-122-5P+ miR-146a-5P+ miR-184+ miR-215-5P+ miR-378d;
